# Production and Purification of Dengue Virus-like Particles from COS-1 Cells

**DOI:** 10.21769/BioProtoc.3280

**Published:** 2019-06-20

**Authors:** Jedhan Ucat Galula, Gwong-Jen J. Chang, Day-Yu Chao

**Affiliations:** 1 Graduate Institute of Microbiology and Public Health, College of Veterinary Medicine, National Chung Hsing University, Taichung, Taiwan; 2 Division of Vector-Borne Diseases, Centers for Disease Control and Prevention, US Department of Health and Human Services, Fort Collins, Colorado, USA

**Keywords:** Dengue virus (DENV), Virus-like particle (VLP), COS-1 cell, VLP purification, Sucrose gradient centrifugation

## Abstract

Non-infectious virus-like particles (VLPs) containing dengue virus (DENV) pre-membrane (prM) and envelope (E) proteins have been demonstrated to be highly immunogenic and can be used as a potential vaccine candidate as well as a tool for serodiagnostic assays. Successful application of VLPs requires abundant, and high-purity production methods. Here, we describe a robust protocol for producing DENV VLPs from transiently-transformed or stable COS-1 cells and further provide an easily adaptable antigen purification method by sucrose gradient centrifugation.

## Background

Flavivirus infection results in the production of virion particles as well as non-infectious virus-like particles (VLPs), which are composed of the pre-membrane (prM) and envelope (E) structural proteins but which lack a nucleocapsid (Allison *et al.*, 1995). In cells transfected with plasmid DNA co-expressing the prM and E proteins, these proteins self-assemble and are secreted in the form of VLPs (Hunt *et al.*, 2001; Chang *et al.*, 2003; Galula *et al.*, 2014). These VLPs have structural and physico-chemical features resembling the virion particles (Shen *et al.*, 2018). DENV VLPs have been demonstrated to be an effective immunogen and have the potential as a subunit vaccine (Konishi and Fujii, 2002; Liu *et al.*, 2014; Shen *et al.*, 2018), and as the alternative serodiagnostic antigens replacing virus-infected suckling mice brain preparations (Holmes *et al.*, 2005; Crill *et al.*, 2009; Chao *et al.*, 2019). Here, we provide a detailed protocol for the production and purification of DENV VLPs from COS-1 cells that can be easily adapted and which requires only basic laboratory equipment.

## Materials and Reagents

- VLP antigen production
  1. T-150 cell culture flasks (Corning, catalog number: CLS430825)
  2. 50 ml conical centrifuge tubes (Thermo Fisher Scientific, catalog number: 339652)
  3. 1.5 ml microcentrifuge tubes (ExtraGene, catalog number: TUBE-170-C)
  4. 0.4 cm electrode gap electroporation cuvettes (Bio-Rad, catalog number: 1652088)
  5. 200 μl pipette tips (Quality Scientific Plastics, catalog number: TW 110-N-Q)
  6. 1 ml pipette tips (Gene Chain Scientific, catalog number: T1000AU)
  7. 0.22 μm filter cup (Sartorius, Sartolab bottle-top filter, catalog number: 180C5-E)
  8. COS-1 cell (ATCC, catalog number: CRL-1650)
  9. DENV VLP plasmid expression vector
*
Note: The plasmid vector described in this protocol expresses DENV-2 strain 16681 prM and E structural protein genes and has been previously characterized in detail (Chang et al., 2003; Galula et al., 2014; Shen et al., 2018).
*
  10. COS-1 cell stably expressing DENV-2 VLP (Holmes *et al.*, 2005)
  11. 0.5% Trypan blue (Bio West, catalog number: L0990)
  12. G418 (Geneticin) (Gibco, catalog number: 11811-031)
  13. Dulbecco’s Modified Eagle’s Medium, High Glucose (DMEM) (Gibco, catalog number: 12800-017)
  14. Fetal bovine serum (FBS) (HyClone, catalog number: SH30084.03)
  15. Non-essential amino acids (Gibco, catalog number: 11140-050)
  16. Penicillin/Streptomycin (Gibco, catalog number: 15140-122)
  17. Sodium bicarbonate (Sigma, catalog number: S4019)
  18. SFM4MegaVir (HyClone, catalog number: SH30587.01)
  19. Cholesterol lipid concentrate (Gibco, catalog number: 12531-018)
  20. L-glutamine (Gibco, GlutaMAX-I, catalog number: 35050-061)
  21. Sodium pyruvate (Gibco, catalog number: 11360-070)
  22. Non-essential amino acids (Gibco, catalog number: 11140-050)
  23. Penicillin/Streptomycin (Gibco, catalog number: 15140-122)
  24. Sodium bicarbonate (Sigma, catalog number: S4019)
  25. Growth medium (see Recipes)
  26. Maintenance medium (see Recipes)
  27. 1x Trypsin-EDTA (Gibco, catalog number: 15400-054) (see Recipes)
  28. 1x Phosphate-buffered saline, pH 7.4 (see Recipes)
- VLP antigen purification
  1. 14 x 89 mm Ultra-Clear ultracentrifuge tube (Beckman Coulter, catalog number: 344059)
  2. 25 x 89 mm Polyallomer ultracentrifuge tube (Beckman Coulter, catalog number: 326823)
  3. 100 kDa MWCO centrifugal filter (Millipore, Amicon^®^ Ultra-15, catalog number: UFC910096)
  4. 0.22 μm syringe filter (Sartorius, catalog number: 16532-K)
  5. Flat-bottom 96-well ELISA plates (Nunc, Nunc Maxisorp, catalog number: 442404)
  6. Kimwipes
  7. 1.5 ml microcentrifuge tubes (ExtraGene, catalog number: TUBE-170-C)
  8. 200 μl pipette tips (Quality Scientific Plastics, catalog number: TW 110-N-Q)
  9. 1 ml pipette tips (Gene Chain Scientific, catalog number: T1000AU)
  10. 10 ml pipettes (GeneDireX, catalog number: PC510-0200)
  11. 25 ml pipettes (GeneDireX, catalog number: PC525-0150)
  12. Sucrose (Sigma, catalog number: S0389)
  13. Glycerol (Sigma, catalog number: G5516)
  14. Sodium carbonate (Sigma, catalog number: S7795)
  15. Sodium bicarbonate (Sigma, catalog number: S4019)
  16. Bovine serum albumin (BSA) heat shock fraction (Sigma, catalog number: A7906)
  17. BSA (New England Biolabs, catalog number: B9000S)
  18. Tween-20 (Able-Bio, catalog number: T003-500)
  19. Rabbit anti-DENV-2 VLP hyper-immune serum (produced in-house)
*
Note: Hyper-immune rabbit serum was produced through repeated intramuscular immunization of rabbit with 100 μg of the DENV-2 VLP plasmid expression vector by electrotransfer mediated protocol (Davis et al., 2001). Alternatively, hyper-immune serum can be produced by intramuscular immunization of rabbit with 100 μg of the DNA plasmid followed by repeated subcutaneous boosting with 100 μg of His-tagged DENV-2 strain 16681 E protein ectodomain III (EDIII) that is expressed and purified from E. coli cells.
*
  20. Anti-DENV-2 mouse hyper-immune ascitic fluid (MHIAF)
*Note: MHIAF was obtained from the Diagnostic Reference Laboratory, Division of Vector-Borne Diseases, Centers for Disease Control and Prevention, USA. As antigen detector antibody, this can be substituted with a commercially available flavivirus group cross-reactive 4G2 monoclonal antibody (ATCC, catalog number: VR-1852).*
  21. HRP-conjugated goat anti-mouse IgG (Jackson ImmunoResearch, catalog number: 115-035-062)
  22. 3,3’,5,5’-tetramethylbenzidine (TMB) substrate (Neogen Corp., Enhanced K-Blue TMB, catalog number: 308177)
  23. Sulfuric acid (Sigma, catalog number: 30743-1L-GL)
  24. Bio-Rad protein assay dye reagent concentrate (Bio-Rad, catalog number: 500-0006)
  25. 1x TNE buffer, pH 7.4 (see Recipes)
  26. 60% sucrose (w/v) solution (see Recipes)
  27. 0.1 M carbonate-bicarbonate buffer, pH 9.6 (see Recipes)
  28. 1% (w/v) BSA (see Recipes)
  29. 0.1% (v/v) PBS-T (see Recipes)
  30. 2 N H_2_SO_4_ (see Recipes)

## Equipment

1. 10 ml pipettes (GeneDireX, catalog number: PC510-0200)
2. 25 ml pipettes (GeneDireX, catalog number: PC525-0150)
3. Hemocytometer (Neubauer improved chamber)
4. Pipette filler (Thermo Scientific, S1 Pipet Filler, catalog number: 9511)
5. 20 μl, 200 μl, 1,000 μl pipettes (Denville Scientific, XL3000i)
6. Multichannel pipette (Corning, Costar 12 channels, catalog number: 07-200-132)
7. Class II A2 Biosafety cabinet for tissue culture (*e.g.*, Thermo Fisher Scientific, model: 1300)
8. Humidified 5% CO_2_ incubators set to 37 °C and 28 °C (Thermo Fisher Scientific, Revco Elite II, model: RCO300T-5-ABC)
9. Inverted microscope (Olympus, model: CX41)
10. Water bath
11. Vacuum pump
12. Autoclave
13. Electroporator (Bio-Rad, Bio-Rad Gene Pulser Xcell, catalog number: 165-2660)
14. Microcentrifuge (Thermo Fisher Scientific, Sorvall Legend Micro 17R centrifuge, catalog number: 75002441)
15. High-speed centrifuge (Kubota, Kubota 3740 centrifuge)
16. 50 ml conical tube centrifuge rotor (Kubota, AF-5004CA rotor)
17. Ultracentrifuge (Beckman Coulter, Optima L-90K ultracentrifuge)
18. SW28 rotor (Beckman Coulter)
19. SW41 rotor (Beckman Coulter)
20. 4 °C refrigerator
-80 °C freezer (Thermo Fisher Scientific, Forma -86 °C ULT Freezer, model: 905)
22. Microplate absorbance reader (Tecan, model: Sunrise-Basic Tecan 16039400)

## Procedure

- VLP antigen production
**Method I. Transient expression of DENV VLPs**
DENV VLP antigens can be produced by electroporating COS-1 cells with a plasmid DNA following the previously described protocol with minor modifications (Chang *et al.*, 2000; Chiou *et al.*, 2008).
  1. Culture COS-1 cells in growth medium in 20 T-150 flasks to around 90-95% confluence at 37 °C with humidified 5% CO_2_.
*Note: Passage cells at 1:2 split ratio for at least 2 consecutive days to ensure all cells are actively dividing.*
  2. Decant medium and wash cells with 20 ml of PBS.
  3. Harvest cells by adding 3 ml of warm trypsin per flask. Gently rock back and forth to cover the cell monolayer and remove excess trypsin (leaving approximately 1 ml). Tighten cap and incubate the cells at 37 °C for 2-3 min.
*Note: Harvest cells from a group of five T-150 flasks one at a time.*
  4. Dislodge cells by lightly pounding the side of the flask with hand.
  5. Resuspend cells in 10 ml of growth medium. Gently rinse bottom and sides of flask with medium and collect cells into a 50 ml conical tube on ice.
*Note: Cells are fragile after trypsinization. Treat cells gently during pipetting and keep on ice for the rest of the procedure until electroporation.*
  6. Pellet cells by centrifugation at 400 *× g* and 4 °C for 2 min.
  7. Decant medium and resuspend pellet in 10 ml of ice-cold PBS. Centrifuge at 400 *× g* and 4 °C for 2 min.
  8. Decant supernatant and resuspend pellet in ice-cold PBS at 0.75 ml per flask of cells. Gently pipette up and down the suspension to break cell clumps.
  9. Perform a cell count at 1:100 dilution in 0.5% Trypan blue using a hemocytometer.
  10. Adjust the cell number to 1.5 x 10^7^ cells/ml of ice-cold PBS.
  11. Add 30 μg of plasmid DNA per electroporation in constant volume (≤ 50 μl) into a 1.5 ml microcentrifuge tube.
  12. Gently tap cell suspension and add 500 μl of cells to DNA, then transfer the mixture into an ice-cold 0.4 cm gap cuvette. Avoid bubbles inside the cuvette.
  13. Electroporate cells at 250 V and 975 μF.
*Notes:*
    - *Before proceeding with the samples, first electroporate no DNA, cell only negative controls to determine the proper cell/DNA volume that could achieve a time constant between 28 and 32 ms (30 ms is optimal). Increasing the cell volume decreases time constant.*
    - *Before each electroporation, gently tap the cell suspension and wipe off excess moisture outside the cuvette.*
    - *Leave the cuvettes at room temperature for no longer than 5-10 min before recovering into pre-warmed flasks.*
  14. Recover cells by adding 500 μl of pre-warmed growth medium into the cuvettes and gently pipette the cells using a slender 1 ml pipette tip. Seed the cells from two electroporation reactions into a pre-warmed T-150 flask containing 25 ml of growth medium and disperse cells by gently rocking the flask.
  15. Allow cells to recover for 6-12 h at 37 °C with humidified 5% CO_2_, then replace the growth medium with 50 ml of pre-warmed maintenance medium. Thereafter, transfer the flasks to a humidified 5% CO_2 _incubator set to 28 °C for VLP secretion.
*
Note: It is important to maintain proper pH (7.2) at all time by visually inspecting the color of the medium and testing ~500 μl aliquots with pH paper. Pink-red and yellow-orange colors of the medium indicate pH that is neutral (7.2) and acidic (below 7), respectively (Figures 1A and 1B). When the color of the medium becomes yellow-orange, adjust pH by adding 400-500 μl of 5% sodium bicarbonate solution per flask dropwise until the color of the medium turns back to pink-red.
*
    **Figure 1.**
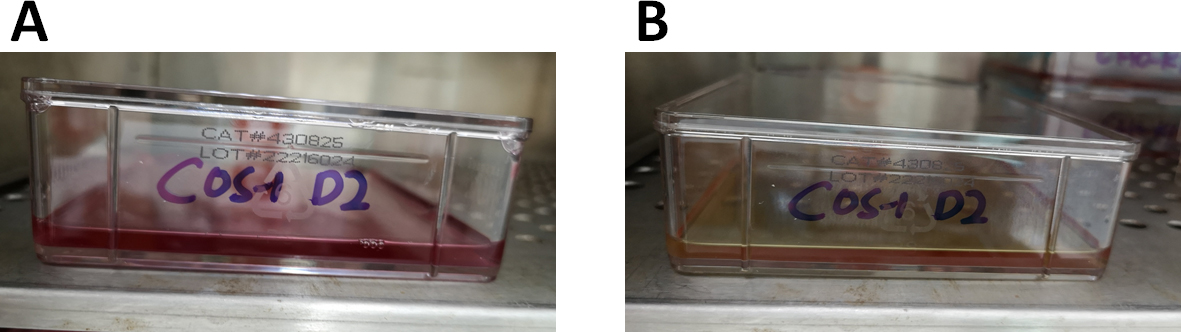
Representative colors of the maintenance medium during VLP antigen production. COS-1 cells maintenance medium is colored red (A) and has a neutral pH of 7.2, which turns to yellow-orange (B) when it becomes acidic (pH below 7).
  16. Harvest culture supernatant 5 days after electroporation and re-feed cells with maintenance medium. Repeat harvest and re-feeding of cells every 5 days thereafter.
*Note: Harvesting and re-feeding of medium can be done at least 4-6 times as long as the cells are still healthy.*
  17. Clarify harvested culture supernatant by centrifugation at 10,000 *× g* and 4 °C for 30 min, and store at 4 °C.
*Note: Store the supernatant at -80 °C if it cannot be processed within 2-3 weeks.*
**Method II. Stable expression of DENV VLPs**
Alternatively, antigen production can be carried out by culturing COS-1 cells stably expressing DENV VLPs (Holmes *et al.*, 2005).
  1. Thaw-out 1 vial of liquid nitrogen-preserved cells in a 37 °C water bath. Add into a 50 ml centrifuge tube containing a warm growth medium and centrifuge at 400 *× g* for 2 min.
  2. Decant supernatant and resuspend cells in 25 ml of warm growth medium. Seed cells into a T-150 flask and incubate at 37 °C with humidified 5% CO_2_.
  3. Add G418 at a final concentration of 500 μg/ml into the growth medium the next day.
  4. Expand cells when they reach 90-95% confluency. Decant medium and wash cells with 20 ml of PBS.
  5. Add 3 ml of warm trypsin. Gently rock back and forth to cover the cell monolayer and remove excess trypsin (leaving approximately 1 ml). Tighten cap and incubate the cells at 37 °C for 2-3 min.
*Note: Antigen producing cells are fragile and sensitive to over trypsinization.*
  6. Dislodge cells by lightly pounding the side of the flask with hand.
  7. Resuspend cells in 10 ml of warm growth medium. Gently pipette up and down the suspension to break cell clumps.
  8. Transfer half of the cell suspension into a new T-150 flask. Add more growth medium containing G418 at a final concentration of 500 μg/ml.
  9. Once cells are expanded in 20 T-150 flasks and at 90-95% confluency, replace the growth medium with 50 ml of maintenance medium. Then, transfer the flasks to a humidified 5% CO_2 _incubator set to 28 °C for VLP secretion.
  10. Harvest culture supernatant every 5 days and re-feed cells with maintenance medium.
*Note: Harvesting and re-feeding of medium can also be done at least 4-6 times.*
  11. Clarify and store harvested culture supernatant as described above.
- VLP antigen purification
Carry out VLP antigen purification with an initial sucrose cushion followed by a rate-zonal centrifugation in a 5-25% linear sucrose gradient (Hunt *et al.*, 2001).
**Day 1**
  1. To ensure sufficient amount of antigen will be purified, concentrate 1 L of the harvested culture supernatant containing the VLPs using a 100 kDa MWCO filter column by centrifugation at 5,000 *× g* and 4 °C.
*Note: Centrifuge the sample for an initial period of 30 min. Thereafter, increase the amount of time needed to concentrate the supernatant to approximately 50-fold of the original volume. Keep concentrated supernatant at 4 °C for purification the next day.*
**Day 2**
  - Filter concentrated supernatant using a 0.22 μm syringe filter.
  - Dilute 60% (w/v) sucrose solution to 20% solution in TNE buffer and filter sterilize.
  - Add 15 ml of 20% sucrose solution into a 25 x 89 mm ultracentrifuge tube, and layer 20 ml of the concentrated supernatant using a 25 ml pipette, then add 2-3 drops (80-100 μl) of 100% glycerol using a 10 ml pipette (Figure 2A).
*Notes:*
    - *The addition of glycerol helps prevent the aggregation and fusion of concentrated VLPs.*
    - *Be careful not to mix the sucrose solution and supernatant. Tilt the tube at 35° angle and set the pipette filler dispense speed to half and partially depress the trigger for slow gravity dispensing.*
    **Figure 2.**
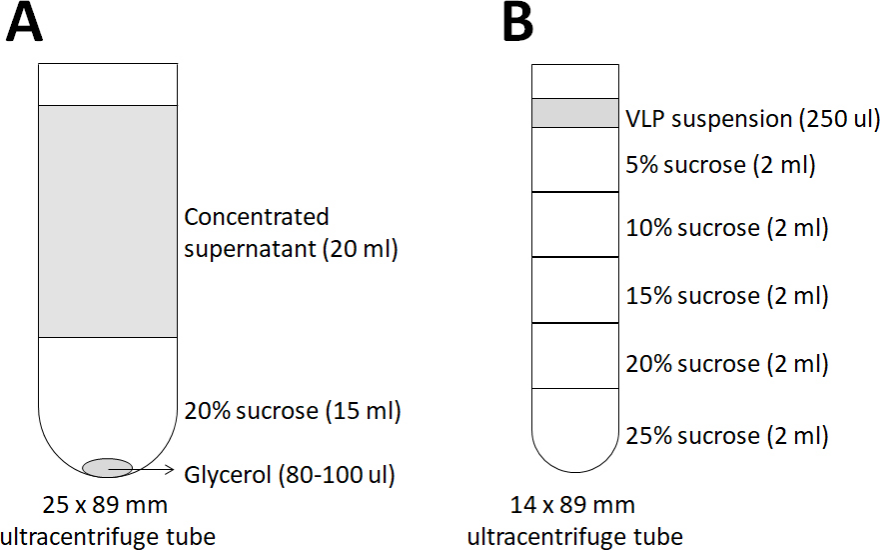
Schematic diagram of the ultracentrifuge tubes for VLP sucrose cushion and sucrose gradient centrifugations. A. For sucrose cushion centrifugation, an SW28 rotor tube (25 x 89 mm) is first loaded with 15 ml of 20% sucrose solution as cushion and layered with 20 ml of the concentrated supernatant, then 80-100 μl of 100% glycerol is added. B. For sucrose gradient centrifugation, a sucrose gradient is manually prepared in an SW41 rotor tube (14 x 89 mm) by carefully layering 2 ml of 25% sucrose solution followed by 20%, 15%, 10%, and 5% sucrose solutions and allowed to mix overnight before loading the VLP suspension prepared from the sucrose cushion centrifugation.
  - Centrifuge the sample at 140,000 *× g* and 4 °C for 16 h using a Beckman SW28 rotor.
**Day 3**
  - Decant sucrose solution and invert tube on Kimwipes for 5-15 min to wick off excess solution.
  - Add 250 μl of TNE buffer to the pellet. Cover the tube with Parafilm and allow the pellet to soften overnight at 4 °C.
  - Prepare 5%, 10%, 15%, 20%, and 25% (w/v) sucrose solutions in TNE buffer by diluting the 60% (w/v) sucrose solution in TNE buffer and filter sterilize.
  - Manually prepare a 5-25% sucrose gradient by carefully layering 2 ml of each sucrose solution using a wide-bore 1 ml pipette tip into a 14 x 89 mm ultracentrifuge tube (Figure 2B).
*Note: Layer the more dense solutions below the less dense solutions. Cover the tube with Parafilm and stand the gradient undisturbed overnight at 4 °C to allow layers to mix.*
  - Coat ELISA plate with 50 μl per well of rabbit anti-DENV-2 VLP hyper-immune serum diluted at 1:500 in carbonate-bicarbonate buffer. Incubate overnight at 4 °C.
**Day 4**
  - Gently resuspend the VLP and transfer to a 1.5 ml microcentrifuge tube. Centrifuge the solution at 16,000 *× g* and 4 °C for 2 min. Transfer the supernatant to a new microcentrifuge tube and repeat the centrifugation.
  - Carefully layer the VLP suspension on top of the sucrose gradient.
  - Centrifuge the sample at 107,000 *× g*, 4 °C for 3 h using a Beckman SW41 rotor.
*Note:* *Set the rotor at slow acceleration and no brake.*
  - Collect 500 μl fractions from top to bottom using 1 ml pipette tips and keep at 4 °C.
*Note: Change pipette tips for each fraction collection.*
  - Perform an antigen-capture ELISA (Crill *et al.*, 2009; Galula *et al.*, 2014) with the collected fractions to determine the peak optical density absorbance at 450 nm (OD 450 nm).
*
Note: Fractions at peak OD values (Figure 1) contain highly pure VLPs.
*
    - Take out the ELISA plate pre-coated with the capture antibody a day before (Day 3, step 10). Dump coating antibody and tap plate thoroughly on paper towels. Add 200 μl per well of 1% BSA and block the plate for 1 h at 37 °C.
    - Dump out the blocking buffer and tap plate thoroughly on paper towels. Add 50 μl per well of each collected fraction diluted at 1:50 in blocking buffer. Incubate the plate for 1 h at 37 °C.
    - Wash the plate with 200 μl per well of 0.1% PBS-T five times. Tap the plate thoroughly on paper towels.
    - Add 50 μl per well of anti-DENV-2 MHIAF diluted at 1:2,000 in blocking buffer. Incubate the plate for 1 h at 37 °C.
    - Wash the plate with 200 μl per well of 0.1% PBS-T five times. Tap the plate thoroughly on paper towels.
    - Add 50 μl per well of HRP-conjugated goat anti-mouse IgG diluted at 1:5,000 in blocking buffer. Incubate the plate for 1 h at 37 °C.
    - Wash the plate with 200 μl per well of 0.1% PBS-T ten times. Tap the plate thoroughly on paper towels.
    - Add 50 μl per well of TMB substrate. Incubate at room temperature for 10 min.
    - Add 50 μl per well of 2 N H_2_SO_4 _to stop the reaction.
    - Measure the optical density at absorbance 450 nm using a microplate reader.
  - Pool the peak fractions (fractions 4-7, Figure 3) and add into a new 14 x 89 mm ultracentrifuge tube. Adjust the final volume to 10 ml with TNE buffer, then add 2-3 drops (80-100 μl) of 100% glycerol using a 10 ml pipette.
    **Figure 3.**
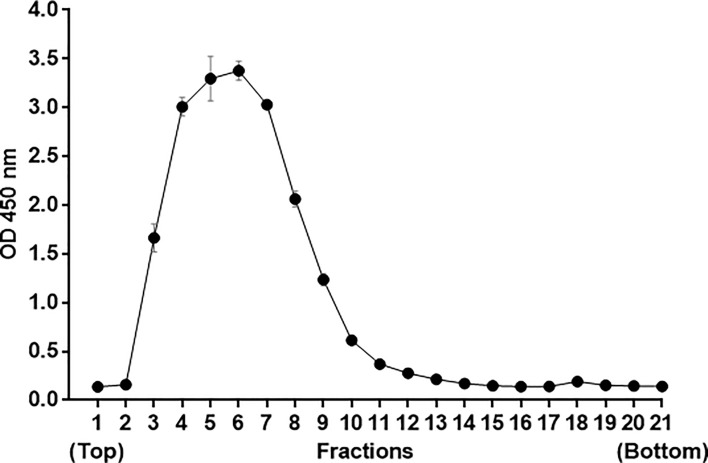
Representative banding profile of DENV2 VLP after rate-zonal centrifugation in a 5-25% linear sucrose gradient at 107,000 *× g* for 3 h. Individual fractions were analyzed by antigen-capture ELISA in duplicates. OD values are means ± SEM of duplicates from a single experiment.
  - Centrifuge the pooled fractions at 270,000 *× g* and 4 °C for 4 h using a Beckman SW41 rotor to pellet the VLPs.
  - Decant sucrose solution and invert tube on Kimwipes for 5-15 min to wick off excess solution.
  - Add 200 μl of TNE buffer to the pellet. Cover the tube with Parafilm and allow the pellet to soften overnight at 4 °C.
**Day 5**
  - Gently resuspend the VLPs and measure the total protein concentration using Bio-Rad protein assay.
  - Aliquot VLPs into several microcentrifuge tubes. Store aliquots at -80 °C until further use.

## Data analysis

Fractions collected after rate-zonal centrifugation in a 5-25% linear sucrose gradient were analyzed by antigen-capture ELISA. The average OD values from duplicates of each fraction were calculated and plotted as an absorbance curve to determine which fractions at peak OD values are to be pooled (fractions 4-7, Figure 3).

## Recipes

1. Growth medium
  1x DMEM, High Glucose  880 ml
  FBS 100 ml
  10 mM non-essential amino acids 10 ml
  100x Penicillin/Streptomycin 10 ml
  Sodium bicarbonate 3.7 g
  Adjust the pH to 7.4. Sterilize using 0.22 µm filter cup
  1x DMEM, High Glucose supplemented to a final concentration of 10% FBS, 0.1 mM non-essential amino acids, 1% Penicillin/Streptomycin, and 3.7 g/L sodium bicarbonate
2. Maintenance medium
  1x SFM4MegaVir 946 ml
  250x Cholesterol lipid concentrate 4 ml
  100x GlutaMAX-I 20 ml
  100 mM sodium pyruvate 10 ml
  10 mM non-essential amino acids 10 ml
  100x Penicillin/Streptomycin 10 ml
  Sodium bicarbonate 2.95 g
  Adjust the pH to 7.4. Sterilize using 0.22 µm filter cup
  1x SFM4MegaVir supplemented to a final concentration of 1x cholesterol lipid concentrate, 4 mM L-glutamine, 1 mM sodium pyruvate, 0.1 mM non-essential amino acids, 1% Penicillin/Streptomycin, and 2.95 g/L sodium bicarbonate
3. 1x Trypsin-EDTA
  Dilute 10 ml of 10x Trypsin-EDTA (0.5%) with 90 ml of 1x PBS
4. 1x Phosphate-buffered saline, pH 7.4
  137 mM NaCl  8.006 g NaCl (MW 58.44 g/mol)
  2.7 mM KCl  0.201 g KCl (MW 74.55 g/mol)
4.3 mM Na_2_HPO_4_  0.765 g NaH_2_PO_4_·2H_2_O (MW 177.99 g/mol)
1.4 mM KH_2_PO_4_  0.191 g KH_2_PO_4_ (MW 136.09 g/mol)
  Dissolve ingredients in double distilled water. Adjust pH to 7.4 and top up the final volume to 1 L
  Autoclave to sterilize
5. 1x TNE, pH 7.4
  50 mM Tris-HCl  7.88 g Tris-HCl (MW 157.6 g/mol)
  100 mM NaCl  5.844 g NaCl (MW 58.44 g/mol)
  0.1 mM EDTA  0.037 g EDTA (MW 372.24 g/mol)
  Dissolve ingredients in double distilled water
  Adjust the pH to 7.4 and top up the final volume to 1 L
  Autoclave to sterilize
6. 60% sucrose (w/v) solution
  Dissolve 300 g of sucrose powder in 1x TNE buffer and adjust the final volume to 500 ml
7. 0.1 M carbonate-bicarbonate buffer, pH 9.6
0.03 M Na_2_CO_3_  1.59 g Na_2_CO_3_ (MW 105.99 g/mol)
0.07 M NaHCO_3_  2.94 g NaHCO_3_ (MW 84.01 g/mol)
  Dissolve ingredients in double distilled water and adjust the final volume to 500 ml. The pH is about 9.6
8. 1% (w/v) BSA
  Dissolve 5 g of BSA heat shock fraction in 1x PBS and adjust the final volume to 500 ml
9. 0.1% (v/v) PBS-T
  Mix 1 ml of Tween-20 in 999 ml of 1x PBS
10. 2 N H_2_SO_4_
Slowly add 28.055 ml of stock H_2_SO_4 _solution to 125 ml double distilled water. Top up the final volume to 500 ml
